# Preliminary Analysis of Genetic Markers for Functional Ethanol Tolerance in Honey Bees (*Apis mellifera*) Using a Free-Flying Paradigm

**DOI:** 10.3390/insects15070494

**Published:** 2024-07-02

**Authors:** Kiri Li N. Stauch, Timothy E. Black, Charles I. Abramson

**Affiliations:** 1 Laboratory of Comparative Psychology and Behavioral Biology, Department of Psychology, Oklahoma State University, Stillwater, OK 74078, USA; 2 Department of Neuroscience & Psychological Sciences, Weber State University, Ogden, UT 84403, USA

**Keywords:** alcohol, *Apis mellifera*, behavior, BKP, HSP70

## Abstract

**Simple Summary:**

The role of two biomarkers, heat shock protein 70 (HSP70) and big potassium ion channel protein (BKP), in the development of functional ethanol tolerance is still being investigated. A study was conducted with honey bees to determine whether differences result at the genetic level for bees that consumed different concentrations of ethanol solutions (i.e., 0%, 2.5%, 5%, or 10%). Additionally, we wanted to know whether the bees behaved differently following their consumption of these different concentrations of ethanol. The bees exhibited changes in their behaviors, but they did not demonstrate differences at the genetic level. Overall, our findings suggest that bees exhibit behavioral changes following ethanol but do not undergo genetic changes at the level that we examined.

**Abstract:**

Honey bees are a commonly used species for alcohol research due to their genome being fully sequenced, their behavioral changes following consumption, and their preference for alcohol. The purpose of this article is to provide a preliminary examination of the genetic expression of heat shock protein 70 (HSP70) and big potassium ion channel protein (BKP) in honey bees following the consumption of either 0%, 2.5%, 5%, or 10% ethanol (EtOH) solutions. The foraging behaviors of the bees were observed and recorded through their return and drinking times. There were significant differences in the return and drinking times between some of the groups. The bees in the 10% condition took significantly longer to return compared to the other groups. Additionally, the bees in the 5% group spent significantly more time drinking compared to the bees in the control (0%) group. There were no significant differences in HSP70 or BKP between the different ethanol groups. Cumulatively, these findings suggest that, while bees may exhibit behavioral differences, the differences in gene expression may not be observed at the transcriptional level.

## 1. Introduction

The three most commonly used species for alcohol research are honey bees, fruit flies (*Drosophilla melanogaster*), and roundworms (*Caenorhabditis elegans*) [1]. These species are ideal for ethanol research due to their exposure to ethanol in their environment, willingness to consume ethanol, and behavioral changes in response to ethanol consumption [1,2,3,4]. Fruit flies have a life stage dependent on alcohol due to hatching in rotting fruit and perform a smaller range of behaviors compared to the honey bee, making the bee a better fit for alcohol studies focusing on a wider array of behaviors [2].

Fruit flies are the primary invertebrate species used for alcohol addiction research [4]. Bees and fruit flies share similarities at the ribonucleic acid (RNA) and protein levels, making them good species for comparative work [5]. Like bees [6,7], fruit flies exhibit similar behavioral changes to humans following ethanol consumption, such as impaired locomotion (e.g., hyperactivity at low doses, loss of balance, and sedation) [8,9]. Previous research suggests that ethanol may impact the ability of a forager bee to find a receiver bee, disrupting her social interaction [10]. However, unlike bees, fruit flies do not live in complex social environments, which does not make them an ideal species for investigating the effects of EtOH on learning and memory [1]. There is evidence that addiction may share pathways with learning and memory, which makes it crucial to have a model species where these pathways can be analyzed [11]. 

Honey bees are an ideal model species for the exploration of the genes related to ethanol tolerance considering the extensive history of ethanol-related laboratory and field research within the species [7,12,13,14]. The honey bee genome has been fully sequenced, which provides clarity when examining the changes in gene expression following ethanol consumption [15]. In naturalistic settings, bees will seek out ethanol in their environment and demonstrate preferences for ethanol solutions [16,17]. Honey bees can come across alcohol while foraging off of fermenting nectar and fruit, inside the hive through yeasts, or through fermented honey in the hive in hot climates [18,19,20]. 

Additionally, bees exhibit behavioral changes in response to ethanol consumption, such as increased aggression [21,22], along with impairments in locomotion [7], learning [23,24], and social communication [25]. Ahmed and colleagues [26] recorded evidence of changes in the body and wing motions in response to incremental ethanol exposure ranging from 0% to 5% ethanol (EtOH). The majority of these changes are dose-dependent, with bees exhibiting impairments in locomotion, learning, and social communication following the consumption of high (5% or above) concentrations of EtOH [7,23,24,25].

The literature on honey bee ethanol research has focused primarily on honey bees in a laboratory setting, with only a few studies focusing on ethanol tolerance in free-flying bees [12,13,27,28]. Stephenson and colleagues [27] found that free-flying honey bees could develop ethanol tolerance over the course of eighteen trips. At concentrations of 2.5% and 5%, the EtOH bees did not exhibit avoidance behaviors; however, at 5%, the EtOH bees exhibited delayed return trips compared to the bees that consumed 2.5% EtOH. Additionally, the bees that received 10% EtOH exhibited higher attrition rates, as measured by non-return to the feeder, in comparison to the bees that received staged increases in EtOH, where they received 2.5%, 5%, and then 10% EtOH [27]. 

Researchers have found that bees that consume concentrations of ethanol that influence their behavior exhibit changes in their gene expression and protein production that are indicative of a stress response [29]. Harnessed bees were fed sucrose solutions containing 0, 2.5, 5, and 10% EtOH. The ethanol-induced increases in expression of HSP70 were measured in the brains of the bees in the 5% EtOH group, providing evidence that the 5% EtOH causes a stress response in the bees’ brains. Furthermore, the bees that consumed 10% EtOH exhibited evidence of an inhibition of the stress response, as indicated by significantly lower HSP70 than the bees that received 5% EtOH [29]. 

In fruit flies (*Drosophila melanogaster*), BKP has been primarily examined as a metric of functional ethanol tolerance through its regulatory gene “slowpoke” (*slo*; [30]). The analysis of the *slo* gene has indicated that its functional products are necessary for the development of rapid ethanol tolerance in fruit flies. In addition, the expression of BKP has been linked to this direct development of ethanol tolerance [31]. It is worth noting that the common approach to assessing the ethanol tolerance in fruit flies is to anesthetize the animals with ethanol vapors and record the recovery time [30,32]. While this is a useful metric, it lacks generalizability across taxa to other species that readily consume ethanol. 

While BKP has primarily been examined within the context of fruit flies, the *slo* gene has been identified in honey bees, along with several of the corresponding ion channel products [33]. In particular, it was noted that the *slo* gene is linked to the production of locomotor behavior in honey bees within the central nervous system. This link, along with the body of literature linking *slo* to rapid ethanol tolerance in fruit flies, makes the gene and its transcriptional products of particular interest to the behavioral expressions of ethanol tolerance. In addition, honey bees have been shown to possess a high degree of genetic conservation when compared to fruit flies [34], making them an ideal organism for the cross-species analyses of the genes primarily studied in the latter organism. Despite this, BKP and *slo* have yet to be analyzed at the transcriptional level in honey bees in conjunction with ethanol tolerance.

The purpose of this study is to provide a preliminary measurement of the effects of ethanol on the expression of the genes linked to ethanol tolerance in honey bees (*Apis mellifera*) using a free-flying paradigm. Specifically, we utilized biomarkers for toxicity and stress by measuring heat shock protein 70 (HSP 70) and big potassium ion channel protein (BKP) [29,31]. These findings will enhance our understanding of the role of genetic markers in the development of functional ethanol tolerance. 

In the present experiment, HSP70 and BKP were examined as potential measurements of ethanol toxicity in honey bees. We anticipated that there would be changes in both biomarkers linked to functional ethanol tolerance. The results from this study may provide information on the use of these biomarkers when they are examined at the transcriptional level. 

## 2. Materials and Methods

Data for this experiment were collected in summer of 2020 in Stillwater, Oklahoma. Bees (*N* = 63) were captured from an artificial feeder containing a 10% sucrose solution using matchboxes. Collecting bees in this manner ensured that all bees were foragers, controlling for individual age within the sample [35]. The matchbox containing the bee was then brought over to a table and placed on a circular platform that contained a drop of 50% sucrose solution. The bee was released from the matchbox with its proboscis extended, which resulted in feeding from the well. Bees were marked while feeding from the well with LBK^TM^ nail polish on the thorax or abdomen when they returned to differentiate them. Once a marked bee returned twice, the test trials started. 

Bees were assigned to one of four ethanol groups, consisting of 0%, 2.5%, 5%, and 10% EtOH and sucrose solutions. For the 10% solution, we used a staged procedure where bees were presented with a small drop of 5% ethanol and sucrose and then the 10% sucrose for the first two test trials. The following eleven test trials consisted of the bee only receiving the 10% ethanol and sucrose solution. This procedure was used for the 10% ethanol solution because the bees would not drink the 10% solution without a transition. The return time (leaving time to next landing time), drinking time (length of consumption; there were starts and stops), and the number of interruptions (if the drinking time was interrupted) were observed and recorded for a total of thirteen test trial visits. Bees dropped from the study if they took longer than 20 min to return between trials. 

The circular platform was cleaned and refilled between each visit. Once bees completed the thirteen trials, they were captured in a matchbox on their fourteenth return. The bees were then placed in a −20 °C freezer until fully sedated and were then transferred into a 1.5 mL PCR tube labeled with the bee’s number. The bees were then placed in a liquid nitrogen tank. Every couple of days, the bees in the liquid nitrogen tank were transported to a −80 °C freezer where they were stored until data collection was completed. 

For analysis of gene expression, a sub-sample (*N* = 24) of each treatment group was taken. All treatment groups were analyzed for normality to ensure no outliers were present or included in the sub-sample. Each subject in the subsequent sample was selected randomly from participants within the behavioral sample. An additional subset of naïve bees (*N* = 6) was collected as genetic controls. These subjects did not receive any experimental manipulation and were collected from the same research hives as experimental subjects. 

Tissue samples were obtained from subjects through brain dissection [29]. Brain tissue was transferred to a 500 µL Trizol solution and homogenized mechanically using a pipette. RNA was obtained from each sample using a Zymo Research Microprep (Quick-RNA Microprep kit, Cat. R1051, Zymo Research, Irvine, CA, USA) column extraction. Initial concentrations of RNA were assessed using a nanodrop, and all samples were subsequently diluted using RNAse free water to an equivalent starting concentration of 20 ng/µL. Samples were stored at −80 °C until analysis.

Cycle threshold data were subject to data transformation following the ddCT method [36]. This method transforms data into the more easily interpreted fold change measure, by comparing expression of genes of interest to that of a housekeeping gene, and to genetically similar subjects that have not undergone experimental manipulation. Data transformation in this way compares expression of genes of interest to a housekeeping gene, which reflects baseline mRNA transcription. For this study, the small ribosomal protein subunit (RS5) was selected as the housekeeping gene. This has been used as a housekeeping gene in past research [37].

In order to quantify relative gene expression, samples were subject to reverse transcriptase quantitative polymerase chain reaction (RT-qPCR). RTqPCR analysis was conducted using a one-step BioRad iTaq Universal SYBR Green qPCR kit (Cat. 172-5151, Bio-Rad Laboratories, Hercules, CA, USA). Master mixes containing reaction reagents and primers for either BKP, HSP70, or RS5 were generated following the procedure for a 10 µL final reaction volume. Mixes contained 5 µL SYBR Green reaction mixture, 0.125 µL iScript reverse transcriptase, 0.15 µL of reconstituted RNA primers for both forward and reverse sequences (See Table 1), and 0.575 µL of nuclease-free H_2_O. Mixes were introduced into each well in 8 µL aliquots, with 2 µL of the respective sample. Each sample was analyzed in triplicate technical replicates, with averages across replicates used for data analysis. Data were recorded using a using a Bio-Rad CFX Connect Real-Time PCR system (Bio-Rad Laboratories, Hercules, CA, USA). Cycles for each plate followed the same cycling procedure, with an initial reverse transcription reaction for 10 min at 50 °C, a polymerase activation phase of 1 min at 95 °C, followed my 40 amplification cycles of 10 s denaturation at 95 °C and extension of 30 s at 60 °C. Cycle threshold data were recorded for each sample and gene.

All data analysis was conducted using the Statistical Package for the Social Sciences (SPSS) software (Version 28). Four one-way analysis of variance (ANOVA) tests were conducted. Two of the analyses were conducted on the behavioral data (i.e., return time and drinking time), while the other two analyses were on the gene expression for HSP70 and BKP.

## 3. Results

**Figure 1:**
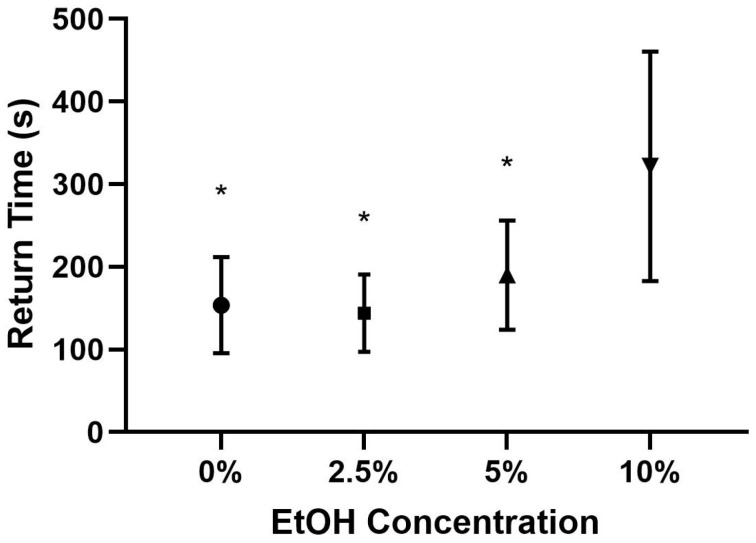
Average return times by EtOH condition with standard deviations. Note: * indicates a statistically significant difference between the group and the 10% group at *p* < 0.05.

The first two one-way ANOVA tests were conducted on the behavioral data, with one test for average return time (*F*(3,62) = 12.793, *p* < 0.001) and a second on average drinking time, *F*(3,62) = 4.127, *p* = 0.010. Tukey HSD post hoc tests were used to conduct pairwise comparisons. The post hoc tests indicate that there was a significant difference in the average return time between the bees in the 10% condition and those in the 0% (*p* < 0.001), 2.5% (*p* < 0.001), and 5% (*p* < 0.001) EtOH conditions; see Table 2 and Figure 1 for the descriptive statistics. The bees in the 10% condition took significantly longer to return compared to the other groups. 

**Figure 2:**
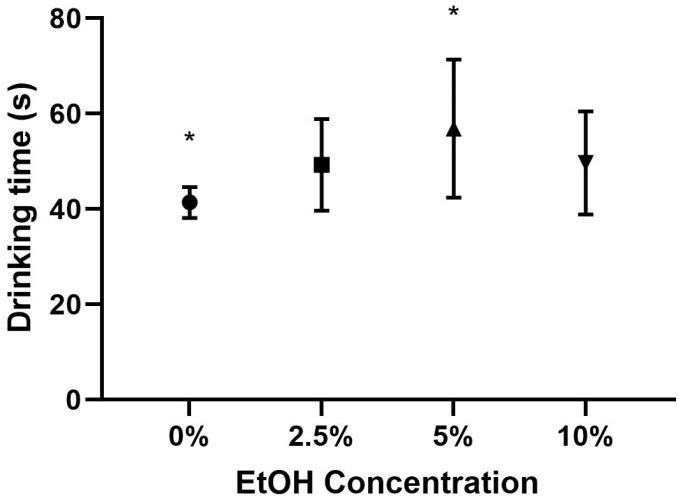
Average drinking times by EtOH condition with standard deviations. Note: * indicates a statistically significant difference between the two groups at *p* < 0.05.

There was a significant difference in the average drinking time between the 0% and 5% conditions, *p* = 0.005; see Table 2 for the descriptive statistics. The bees in the 5% condition spent significantly longer drinking compared to the bees in the 0% condition. There was not a significant difference in drinking time between the bees in the remaining pairs, 0%, 2.5%, 5%, and 10%; see Table 2 and Figure 2 for the descriptive statistics. 

**Figure 3:**
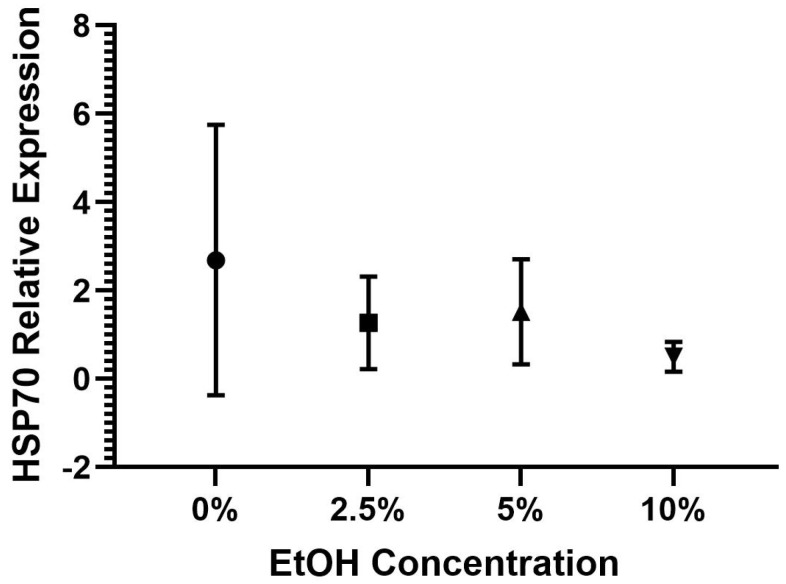
Average HSP70 expression by EtOH condition with standard deviations.

**Figure 4:**
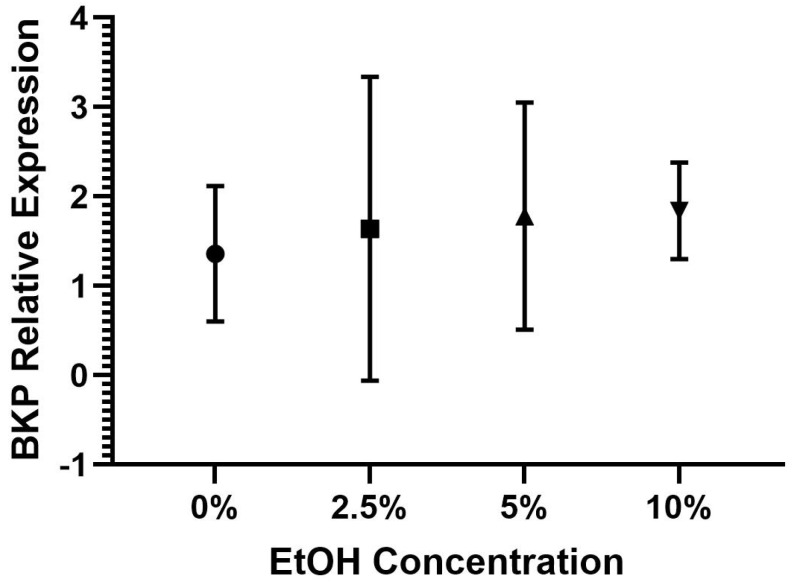
Average BKP expression by EtOH condition with standard deviations.

The one-way ANOVAs for HSP70 (*F*(3,20) = 1.82, *p* = 0.176) and BKP (*F*(3,20) = 0.90, *p* = 0.460) were not significant. The results indicate that there was not a significant difference in the HSP70 expression between the bees in the 0%, 2.5%, 5%, and 10% EtOH conditions; see Table 3 and Figure 3 for the descriptive statistics. There was also not a significant difference in the BKP expression between the bees in the 0%, 2.5%, 5%, and 10% EtOH conditions; see Table 3 and Figure 4 for the descriptive statistics. Combined, these findings suggest that the bees in the different EtOH conditions and the control group (0% EtOH) did not express significantly different levels of HSP70 and BKP; see Table 3 for the descriptive statistics. 

## 4. Discussion

Overall, the bees exhibited some significant differences in foraging behavior, as suggested by their return times and drinking times. The bees did not exhibit significant differences in the HSP70 and BKP gene expressions. Combined, these results suggest that, although we observed behavioral changes following some concentrations of EtOH, we did not witness a corresponding change in mRNA expression. 

There were significant differences in return time for the bees in the highest EtOH condition (10%) compared to the 0%, 2.5%, and 5% conditions. The bees in the 10% group took significantly more time to return. Similarly, Bozic et al. [25] found that the bees in their 10% EtOH condition visited a feeder less frequently and spent more time in their hive. Additionally, they observed that the bees exhibited difficulty flying [25]. During the study trials, the researchers observed the bees in the 10% condition exhibiting impaired locomotion. The bees in this group had difficulty landing and would sometimes fall over during landing or stumble upon landing. Also, the bees would have difficulty taking off and appeared to have difficulty circling while leaving. Abramson and colleagues [6] also found a significant decrease in locomotion following the consumption of a 10% EtOH solution, providing evidence of motor impairment.

In addition to the differences in return time, the bees differed in their drinking times. Specifically, the bees in the 5% condition spent significantly longer drinking compared to the bees in the 0% condition. It is likely that the bees in the 0% and 2.5% groups did not suffer from locomotor impairments and were able to feed more easily. The bees in the 2.5% group had the quickest average return time, followed by the bees in the control, 5%, and 10% groups, respectively. We believe that the bees in the 2.5% group may have exhibited hyperactivity, as evidenced by their return time.

Exposure to 2.5% EtOH or 5% EtOH has been linked to changes in honey bee flight kinematics [26]. Researchers found that the bees in both the 2.5% and 5% EtOH groups exhibited a frequency decrease and amplitude increase in wing strokes in bees that were hovering in flight [26]. The bees that consumed a 10% EtOH sucrose concentration did not initiate flight during the study. The bees in the 2.5% and 5% EtOH groups were also observed exhibiting erratic locomotion while on the ground [26].

The results from our study are similar to some of those that Ahmed and colleagues [26] found. The bees in our study exhibited issues with flight in the 10% group, although they did not fail to take off. The bees in our 2.5% and 5% groups walked around quite a bit during the study but did not exhibit issues with righting themselves like the 10% group.

The bees in the 10% condition had difficulty with motor coordination, as evidenced by their return times, which may account for their lack of change in drinking time from the other groups. During the study, the bees in the 10% condition were observed having difficulty landing and would land off balance and be unsteady upon take off at later visits. These changes were not noticed in the bees in the lower alcohol concentration groups. Meanwhile, while the bees in the 5% group did not exhibit decreased motor function, they may have exhibited increased hyperactivity, although not enough to cause a significant difference in return time. Maze and colleagues [7] reported that bees that consumed 5% EtOH showed increases in walking time, suggesting hyperactivity.

The results of the RT-qPCR assay indicated that there were no significant differences in the transcriptional expression of either HSP70 or BKP. At first glance, these data appear to be counter to the past literature on both genes, indicating that the expression of both should be altered by the presence of ethanol. Hranitz et al. [29] noted increases in HSP70 expression following ethanol ingestion in honey bees; however, this increase was recorded at the protein level. It is possible that the changes ethanol consumption exerts on HSP70 expression occur at the post-transcriptional level. This would explain the increase in expression observed by Hranitz et al. [29], while no such change was observed in the present study. Past studies have examined HSP70 as a metric of cellular oxidative stress using RT-qPCR [37], and have also noted relatively little change in gene expression as a result of experimental manipulation. This corroborates the hypothesis that a change in HSP70 as a result of extra-organismal factors is likely an effect exerted at the post-transcriptional or even post-translational level. Further, Johnston et al. [38] have noted that, in response to external stressors, the expression of mRNA transcription factors can be altered, subsequently altering the transcription, gene splicing, and even protein folding. The particular gene in question, *Xbp1*, is linked to the overall changes in the proteins responsible for the cellular immune responses in honey bees. While Johnston et al. [38] did not link this transcription factor directly to HSP70, the role of the protein as a measure of oxidative cellular stress suggests that it could be subject to this, or a similar regulatory factor, triggered by the manipulations present in the present work.

In addition to the manipulations present in this study, it is important to note that this is a free-flight paradigm. Such designs enable a wide variety of behavioral outcomes on the part of subjects. While this does generate more generalizable data to real-world scenarios, it also reduces the level of experimental control concerning the amount of ethanol ingested by each individual. This lack of direct ethanol quantification may drive the non-significant results observed in the present experiment. The study by Hranitz et al. [29] made use of a direct feeding methodology, administering a set amount of solution. The methodological differences between these two studies may also contribute to the differences in our results.

In the case of BKP, this is the first work examining BKP as an isolated portion of the *slo* gene within honey bees. The *slo* gene itself codes for several proteins, including various isoforms of BKP. It is likely that the specific mRNA strand analyzed in this current work is a portion of the *slo* gene that is not necessary for the development of ethanol tolerance, and as such did not alter the expression following ethanol ingestion. Further, while well-studied in *drosophila*, there is relatively little research regarding the *slo* gene within honey bees. Despite their genetic similarity [33,34], it is possible that the *slo* gene’s role within honey bees is more locomotor and less implicated in the development of ethanol tolerance.

Further research is necessary regarding the genetic components of ethanol tolerance. We suggest that researchers explore the *slo* gene in the honey bee to determine whether it plays more of a role in locomotion compared to developing tolerance to ethanol. Further, researchers should conduct studies examining HSP70 and BKP in honey bees following chronic exposure. The bees in our experiment were used in one session, so, while they received alcohol throughout their trials, they did not receive alcohol several days in a row. Overall, our experiment provides information suggesting that both HSP70 and BKP might not play a role in the alcohol tolerance in honey bees at the transcriptional level.

## Figures and Tables

**Table 1:** mRNA primer sequences for genes of interest (BKP and HSP70) and housekeeping gene (RS5) for RT-qPCR reactions.

Gene	Forward Primer Sequence (5′-3′)	Reverse Primer Sequence (5′-3′)
HSP70	GTGATGAACGATGGAGGAAA	GAAATARGCTGGGACGGTAATA
BKP	CACTAGGCCCCCGTCGTCTCT	GGCAGCCACTCCCGCGATGT
RS5	TAACGTCCAGCAGAATGTGGTA	AATTATTTGGTCGCTGGAATTG

**Table 2:** Descriptive statistics for average return time and drinking time by EtOH condition.

EtOH Concentration	Return Time *M*	Return Time *SD*	Drinking Time *M*	Drinking Time *SD*
0%	153.74	58.29	41.40	3.26
2.5%	144.03	46.73	49.33	9.60
5%	190.15	66.04	56.88	14.47
10%	321.72	138.99	49.70	10.77

Note: *M* is mean and *SD* is standard deviation.

**Table 3:** Descriptive statistics for HSP70 and BKP.

EtOH Concentration	HSP70 *M*	HSP70 *SD*	BKP *M*	BKP *SD*
0%	2.69	3.06	1.36	0.76
2.5%	1.27	1.05	1.64	1.70
5%	1.52	1.19	1.78	1.27
10%	1.19	0.34	1.84	0.54

Note: units for genetic expression are in fold change (FC). *M* is mean and *SD* is standard deviation.

## Data Availability

The data are available upon request.
